# 

**Published:** 1896-06

**Authors:** 


### CONTENTS.

ORIGINAL ARTICLES:

On Intestinal Parasitism in the Dog, and its Treatment. By CECIL FRENCH, D.V.S. 441

Treatment of Actinomycosis. By DR. V. SCHAEFER . . . . . . . . 452

Hereditary Transmission of Tuberculosis. By S. H. WARD, V.S. . . . 456

A Plea for the Institution of a New Special Course of Instruction in Veterinary Colleges. By SAMUEL GOLDSTEIN, A.B., M.D., . . . . . . . . 459

Tuberculosis in Chickens. By T. A. BRAY, D.V.S. . . . . . . . 461

Notes on the Nodes Found in the Lungs Caused by Actinomyces Bovis, Micrococcus Botryogenus, Strongylus, Echinococci, and Aspergillus. By J. T. GLENNON . 462

Sporadic Pleuro-pneumonia. By F. A. THOMAS, V.S. . . . . . . . 464

ABSTRACTS FROM FOREIGN JOURNALS:

Observations in Regard to the Application of Carbolic Acid—Cattle-legislation in Austria—Empirics in Austria—Laws for Stamping Out Cattle-Tuberculosis in France—Increase in the Number of Military Veterinary Surgeons—For the Prevention of Mistakes in the Handling of Medicines—Tetanus-cure by a Sudden Shock to the Nerves—Stupefying Animals Before Slaughter—Bacteriological Course for Veterinarians in Baden—Two Cases of Actinomycosis Treated with Iodcalium—Periodical Lameness in the Horse—Tuberculosis at the International Veterinary Congress—Celebration of the Belgian Veterinary Federation in Honor of Professor Alph. Degive, State Director of the School of Veterinary Medicine . . . . . . . . . . . . . 467–477

REPORTS OF CASES:

An Ingenious and Useful Device. By D. MCNAUGHT, V.S. . . . . . . 477

Ascaris Megalocephalis in the Hog. By A. YOUNGBERG, D.V.S., . . . 478

EDITORIAL:

Veterinary Journalism—The Danger of Living in Glass Houses—What Will the Others Do?—Canadian Diplomacy and our Cattle-exports—Defeat Senate Bill 1552—The New York Registry Bill . . . . . . . . . 480–483

CORRESPONDENCE . . . . . . . . . . . . . . . 484

CONTROL WORK:

Pennsylvania—Virginia—Missouri—Connecticut—Nebraska—Kansas—Texas—Indiana—Massachusetts—New Jersey . . . . . . . . . 485–488

COMMENCEMENT EXERCISES:

Detroit Veterinary Medical College—American Veterinary College—National Veterinary College . . . . . . . . . . . . . . 488–489

SOCIETY PROCEEDINGS:

Keystone Veterinary Medical Association—Michigan State Board of Health—Veterinary Medical Association of New Jersey—New York County Veterinary Medical Association—New Hampshire Veterinary Medical Association—Missouri Valley Veterinary Association—Important Meetings in June—McGill Society for Study of Comparative Psychology—Manitoba Veterinary Medical Association 490–503

PERSONAL—GLEANINGS—NEW INVENTIONS—WHAT WE WANT TO KKOW . 503–508

